# Cardiovascular determinants of resuscitation from sepsis and septic shock

**DOI:** 10.1186/s13054-019-2414-9

**Published:** 2019-04-15

**Authors:** Fabio Guarracino, Pietro Bertini, Michael R. Pinsky

**Affiliations:** 1 Department of Anesthesia and Critical Care Medicine, Azienda Ospedaliero Universitaria Pisana, Pisa, Italy; 2 Department of Critical Care Medicine, University of Pittsburgh, 1215.4 Kaufmann Medical Building, 3471 Fifth Avenue, Pittsburgh, PA 15213 USA

**Keywords:** Heart-lung interactions, Blood volume, Ventriculo-arterial coupling, Effective circulating blood volume, Norepinephrine, Clinical trial

## Abstract

**Background:**

We hypothesized that the cardiovascular responses to Surviving Sepsis Guidelines (SSG)-defined resuscitation are predictable based on the cardiovascular state.

**Methods:**

Fifty-five septic patients treated by SSG were studied before and after volume expansion (VE), and if needed norepinephrine (NE) and dobutamine. We measured mean arterial pressure (MAP), cardiac index (CI), and right atrial pressure (Pra) and calculated pulse pressure and stroke volume variation (PPV and SVV), dynamic arterial elastance (Ea_dyn_), arterial elastance (Ea) and left ventricular (LV) end-systolic elastance (Ees), Ees/Ea (VAC), LV ejection efficiency (LVeff), mean systemic pressure analogue (Pmsa), venous return pressure gradient (Pvr), and cardiac performance (Eh), using standard formulae.

**Results:**

All patients were hypotensive (MAP 56.8 ± 3.1 mmHg) and tachycardic (113.1 ± 7.5 beat min^−1^), with increased lactate levels (lactate = 5.0 ± 4.2 mmol L^−1^) with a worsened VAC. CI was variable but > 2 L min^−1^ M^−2^ in 74%. Twenty-eight-day mortality was 48% and associated with admission lactate, blood urea nitrogen (BUN), and creatinine levels but not cardiovascular state. In all patients, both MAP and CI improved following VE, as well as cardiac contractility (Ees). Fluid administration improved Pra, Pmsa, and Pvr in all patients, whereas both HR and Ea decreased after VE, thus normalizing VAC. CI increases were proportional to baseline PPV and SVV. CI increases proportionally decreased PPV and SVV. VE increased MAP > 65 mmHg in 35/55 patients. MAP responders had higher PPV, SVV, and Ea_dyn_ than non-responders. NE was given to 20/55 patients in septic shock, but increased MAP > 65 mmHg in only 12. NE increased Ea, Ea_dyn_, Pra, Pmsa, and VAC while decreasing HR, PPV, SVV, and LVeff. MAP responders had higher pre-NE Ees and lower VAC. Dobutamine was given to 6/8 patients who remained hypotensive following NE. It increased Ees, MAP, CI, and LVeff, while decreasing HR, Pra, and VAC. At all times and all steps of the protocol, CI changes were proportional to Pvr changes independent of treatment.

**Conclusions:**

The cardiovascular response to SSG-based resuscitation is highly heterogeneous but predictable from pre-treatment measures of cardiovascular state.

**Electronic supplementary material:**

The online version of this article (10.1186/s13054-019-2414-9) contains supplementary material, which is available to authorized users.

## Introduction

Human sepsis is the result of a complex pathological process characterized by an infection-induced generalized intravascular inflammatory state causing marked dysregulation of cardiovascular adaptive responses [1, 2]. Initial management, as defined by the Surviving Sepsis Guidelines (SSG), focuses on source control, early broad-spectrum antibiotics, and cardiovascular stabilization [3]. Systemic hypotension often persists following initial volume expansion (VE), independent of cardiac index (CI) characterizing generalized vasoplegia and ventricular dysfunction [2]. These complex and changing processes make the cardiovascular management of septic shock patients not only difficult but preclude simple formulaic approaches. Importantly, the fundamental pathological states defining human septic shock and its cardiovascular response to resuscitation therapies have not been described together. For example, levels of volume responsiveness, cardiac contractility, and ventriculo-arterial coupling (VAC) prior to resuscitation have been described, but not in the same subjects. Since there is much heterogeneity in individual patient cardiovascular reserve, we reasoned that there would be great value in linking desperate measures from different validated techniques into a common mythological approach and then applying a fixed generally recognized resuscitation protocol within this context. Intravascular fluid infusion to restore an adequate pressure gradient for venous return, vasopressor-induced increased vasomotor tone to maintain organ perfusion pressure, and inotropes to improve cardiac contractility represent the three primary cardiovascular therapies used to restore cardiovascular stability in septic shock patients [3]. A more efficient and effective personalized resuscitation would greatly benefit from such an understanding of the patient’s initial cardiovascular state, reserve, and response to resuscitation efforts.

We and others have documented the ability to estimate at the bedside of patients in septic shock mean systemic pressure (Pms) [4], left ventricular (LV) end-systolic elastance (Ees) [5], arterial elastance (Ea), and their associated derived cardiovascular parameters, allowing a deeper understanding of cardiovascular status and response to therapy. For example, based on Guytonian physiology, for cardiac output (CO) to increase, the pressure gradient for venous return (Pvr) (Pms minus right atrial pressure (Pra)) must increase [6], the resistance to venous return decrease [7], or both. Similarly, global cardiac efficiency (Eh) is quantified as the ratio of Pvr to Pms, with the more efficient heart increasing Pra less with fluid administration as CO increases [8]. Furthermore, the ratio of Ea to Ees, defining VAC [9], independently predicts outcomes in patients with cardiovascular disease [10] and is impaired in vasoplegic [11] and septic shock patients [12], suggesting that some of the septic myocardial depression reflects decoupling [13]. VAC is tightly linked to measures of LV ejection efficiency (LVeff), defined as the ratio of external work to total cardiac work during one cardiac cycle and is considered a major determinant of cardiac performance, being impaired if the Ea/Ees ratio varies too far in either direction from normal. Also, the dynamic measures, such as pulse pressure variation (PPV) or stroke volume variation (SVV), predict volume responsiveness [14], and the ratio of PPV to SVV, termed dynamic arterial elastance (Ea_dyn_), predicts the blood pressure response to changing cardiac output [15] or decreasing norepinephrine [16]. Thus, bedside clinicians have available to them an impressive array of potential cardiovascular diagnostic tests to define the cardiovascular state and reserve of their patients as well as accurately predict their response to specific therapies.

Although these powerful methods have been used separately to assess selected cardiovascular aspects of septic shock resuscitation, their combined analysis during protocolized resuscitation steps in septic patients is lacking. Thus, we analyzed the cardiovascular effects of standardized SSG resuscitation protocol [3] in septic patients. We hypothesized that the cardiovascular responses to resuscitation are predictable based on the cardiovascular state as quantified by these measured cardiovascular variables.

## Methods

After ethical committee for human experimentation approval of both institutions, we enrolled 55 septic patients treated according to SSG [3]. Entry criteria included all patients sequentially presenting with presumed sepsis during the hours when the investigators (FG, PB) were available. Sepsis was defined as a probable infectious etiology, hypotension (mean arterial pressure (MAP) < 65 mmHg), and lactic acidosis (lactate > 2 mmol dL^−1^). All patients who became stable signed informed consent, whereas those who never regained consciousness did not sign the consent but, as allowed by EEC rules, were still included in the analysis. We excluded patients < 18 years of age and those patients with known significant mitral valve regurgitation and aortic valve or aortic pathologies because the arterial pressure signal was to be used to estimate CO.

Initial cardiovascular management included sequential VE with 30 mL/kg 0.9 N NaCl within < 3 h of enrollment, followed by norepinephrine infusion (NE), if needed to achieve an initial MAP > 65 mmHg following VE, titrated between 0.01 and 1 μg kg^−1^ min^−1^, and then inotropic support with dobutamine was titrated between 5 and 15 μg kg^−1^ min^−1^, if evidence of hypotension and organ hypoperfusion persisted following NE, with all treatments given within < 5 h after enrollment. We limit norepinephrine to 1 μg kg^−1^ min^−1^ to minimize the deleterious effects of high-dose vasopressors. All resuscitation decisions were made by the attending physicians. All patients had blood samples, appropriate body fluid, and other appropriate source cultures taken and received broad-spectrum antibiotics within 2 h of presentation and required mechanical ventilation. Prior to the initiation of the initial 30 mL kg^−1^ fluid bolus, we estimate that patients received between 250 and 500 mL 0.9 N NaCl during the initial instrumentation.

Patients were instrumented with a central venous catheter, to infuse fluids and drugs and to measure Pra, a radial artery catheter to monitor MAP, to measure systolic arterial pressure, and by uncalibrated pulse contour analyses (MostCare®, Vygon, Italy) to continuously estimate CI, LV stroke volume, SVV, and PPV. Transthoracic echocardiography (TTE) was performed upon admission and then after each therapeutic intervention, if needed. Transesophageal echocardiography was used when TTE did not give sufficient images to assess cardiac function. Echocardiographic estimates of CO were used to assess the accuracy of the Mostcare® CO measures across treatments. Electrocardiograph (lead 2) RR intervals defined heart rate (HR) and peripheral pulse oximetry pulse oxygen saturation. Controlled mechanical ventilation was at a tidal volume of 6–8 mg kg^−1^ ideal body weight with a positive end-expiratory pressure/FiO_2_ ratio as defined by ARDSnet guidelines [17]. Respiratory frequency was adjusted to end-tidal CO_2_ between 32 and 35 mmHg.

**Fig. 1:**
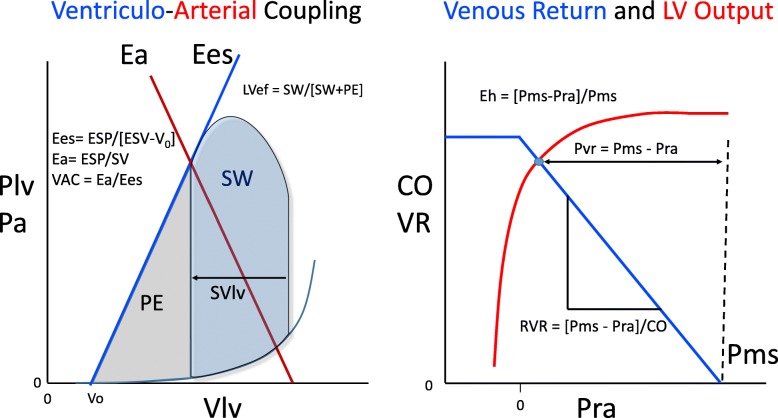
Left panel: A stylized representation of the relation between left ventricular (LV) pressure (Plv) or arterial pressure (Pa) and both the LV pressure-volume relations during a cardiac cycle and arterial elastance (Ea) (red line) along with the associated formulae defining end-systolic elastance (Ees) (blue line) and Ea. Stroke work (SW) is the area within the LV pressure-volume loop for one cardiac cycle, while the potential energy (PE) is the area sub served by the Ea and LV end-systolic volume (ESV). LV efficiency (LVef) is the ratio of SW to SW + PE. Right panel: A stylized representation of the relation between steady-state cardiac output (CO) or venous return (VR) and right atrial pressure (Pra) from the perspective of venous return (blue line) and left ventricular output (red line) illustrating global cardiac efficiency (Eh). The zero CO pressure intercept defines mean systemic pressure (Pms). See text for further discussion

Derived hemodynamic parameters were calculated using standard formulae as illustrated in Fig. 1 and described in the Additional file 1: Methods. Briefly, LV Ees was calculated by the single beat method of Chen et al. and Ea as the ratio of 0.9∙systolic arterial pressure and SV [5]. Ees zero pressure intercept defined Vo, and the potential energy (PE) per beat defined by the area of the Ees to end-systolic volume triangle [18]. LV stroke work (SW) was the area inside the LV pressure-volume loop estimated as the product of end-systolic pressure (ESP) and SV. LV pressure-volume area (PVA) was the sum of PE and SW, and the SW to PVA ratio defining LVeff [18]. Analogue Pms (Pmsa) was derived from paired end-expiratory CO, Pra, and MAP measures by the method of Parkin and Leaning [8], and (Pmsa-Pra)/Pmsa defining cardiac performance (Eh). End-systolic and end-diastolic volumes (ESV and EDV) were measured by echocardiography. All echocardiographic measures were made by clinicians (FG, PB) expert in single beat Ees and Ea estimates.

### Statistical analysis

To obtain a statistical power of 0.8 with alpha 0.5 and 95% confidence intervals for the first enrollment were calculated a priori that we would need 42 patients [12]. Thus, we recruited over 50 patients to overcome any a priori un-estimated biases. All data was tested for normality using Shapiro-Wilk analysis, and paired sample *t* test and Wilcoxon ranked sign test were carried out to assess differences induced by interventions. Independent sample *t* test for normally distributed data and Mann-Whitney *U* test for nonparametric measurements were used to test differences between variables grouped by MAP restored (> 65 mmHg), MAP increased, and CI increased at ≥ 15%. Pearson’s correlation analysis was used to find correlation between normally distributed data, Spearman’s correlation was used for nonparametric data, and chi-square test was used to find correlation between categorical data. Receiver operating characteristic analysis was used to test sensitivity and specificity of hemodynamic variables in predicting changes in categorical endpoints. Statistical studies were two-tailed, and *p* value of less than 0.05 was considered significant. Analyses were performed using SPSS (v23 for Mac, IBM USA). Unless otherwise stated, data are presented as mean ± SD.

## Results

### Baseline pre-resuscitation status

Patient features on admission are described in Table 1. All were hypotensive (MAP 56.8 ± 3.1 mmHg) and tachycardic (113.1 ± 7.5 beats min^−1^), with elevated lactate levels (lactate 5.0 ± 4.2) and had an increase in Ea relative to Ees consistent with ventriculo-arterial uncoupling. CI was variable, but in 74%, CI was > 2 L min^−1^ M^−2^. The mean admission SOFA score was 16.1, and the 30-day mortality rate was 47%. Thirty-day mortality was associated with higher admission lactate, BUN, and creatinine levels, but not with any other measured or derived hemodynamic variables. Specifically, 35 patients resolved their hypotension with VE alone but retained a 44% 30-day mortality rate, whereas those in septic shock had a 52% 30-day mortality rate of which was not significantly different. Additional file 2: Table S1 lists individual patient sepsis etiologies, SOFA and SAP II scores, and maximal doses of norepinephrine and dobutamine given.

**Table 1:** Demographics of group on admission and relation to survival at 30 days

Group variable	Total (mean ± SD)	Alive at 30 days (mean ± SD)	Dead at 30 days (mean ± SD)	*p*
*N*	55	29 (53%)	26 (47%)	
Age (years)	69 ± 11	69 ± 12	71 ± 11	ns
Gender	M 34, F 21	M 9, F21	M 10, F 10	ns
MAP (mmHg)	57 ± 3	57 ± 3	58 ± 2	ns
CI (L min^−1^ M^2^)	2.09 ± 0.11	2.10 ± 0.10	2.04 ± 0.11	ns
HR (min^−1^)	113 ± 8	112 ± 8	114 ± 6	ns
Ea (mmHg mL^−1^)	2.11 ± 0.41	2.10 ± 0.36	2.15 ± 0.48	ns
Ees (mmHg mL^−1^)	1.42 ± 0.35	1.36 ± 0.37	1.54 ± 0.33	ns
Ea/Ees	1.56 ± 0.41	1.67 ± 0.43	1.44 ± 0.39	ns
Pra (mmHg)	7.6 ± 1.4	7.5 ± 1.5	7.6 ± 1.3	ns
Pmsa (mmHg)	13.0 ± 1.4	12.9 ± 1.6	12.9 ± 1.3	ns
Pvr (mmHg)	5.3 ± 0.5	5.3 ± 0.5	5.3 ± 0.5	ns
Eh (%)	0.41 ± 0.05	0.42 ± 0.05	0.42 ± 0.05	ns
LV SW (mmHg mL)	2581 ± 900	2842 ± 740	2414 ± 1018	ns
LV efficiency (%)	0.65 ± 0.12	0.65 ± 0.12	0.63 ± 0.13	ns
Lactate (mmol L^−1^)	5.1 ± 4.3	2.7 ± 1.5	7.1 ± 4.8	< 0.005
Hematocrit (%)	30.0 ± 4.3	30.0 ± 4.3	29.9 ± 4.5	ns
Hemoglobin (g dL^−1^)	9.8 ± 1.4	9.8 ± 1.4	9.8 ± 1.4	ns
BUN (mg dL^−1^)	38.8 ± 29.3	27.3 ± 15.2	49.7 ± 35.3	< 0.005
Creatinine (mg dL^−1^)	1.8 ± 1.2	1.3 ± 0.9	2.2 ± 1.3	0.010
Chronic hypertension	26 (47%)	16 (55%)	10 (38%)	ns
SOFA score	16 ± 7	11 ± 5	21 ± 6	< 0.005
SAPS II score	63 ± 15	52 ± 12	74 ± 8	< 0.005

*Abbreviations*: *M* male, *F* female, *MAP* mean arterial pressure, *CI* cardiac index, *HR* heart rate, *Ea* arterial elastance, *Ees* end-systolic elastance, *Pra* right atrial pressure, *Pmsa* mean systemic pressure analogue, *Pvr* pressure gradient for venous return, *Eh* global cardiac efficiency, *LV* left ventricular, *SW* stroke work, *BUN* blood urea nitrogen

**Fig. 2:**
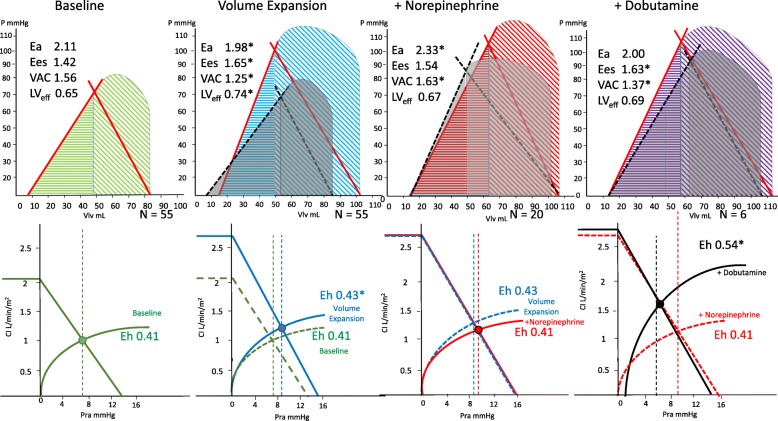
Impact of resuscitation steps on ventriculo-arterial coupling (above) and venous return to cardiac out (below), using Fig. 1 conventions and abbreviations, and reporting the calculated Ea, Ees, VAC, LVef, and Eh for each step. Asterisk connotes a significant change in the value for the group from the previous condition

**Fig. 3:**
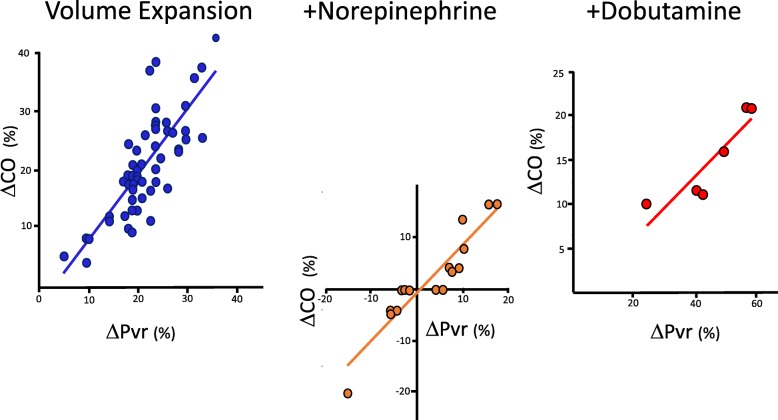
Relation between resuscitation step-induced changes in cardiac output (ΔCO) and changes in the pressure gradient for venous return (ΔPvr) for all subjects receiving volume expansion (blue), norepinephrine (orange), and dobutamine (red), with their associated linear regression lines

Table 2 summarizes the cardiovascular parameters prior to and after the three sequential interventions, and Fig. 2a and b diagrams the associated mean ventriculo-arterial coupling and venous return-LV output changes while Additional file 3: Figure S1-S9 shows individual variables over each step per patient. In all subjects with all treatments, changes in CO co-varied in proportion to changes in Pvr (Fig. 3).

**Table 2:** Cardiovascular effects of resuscitation stages

	Baseline	Volume expansion	Pre-norepinephrine	Norepinephrine	Pre-dobutamine	Dobutamine
Fluid	–	30 mL kg^−1^		–		–
NE	–	–		0.01–1 μg kg^−1^ min^−1^		0.01–1 μg kg^−1^ min^−1^
Dobutamine	–	–		–		5–15 μg kg^−1^ min^−1^
Patients	55	55	20	20	8	8
Time to next step	0	< 3 h		< 5 h		< 5 h
Variable	*x* ± SD	*x* ± SD	*x* ± SD	*x* ± SD	*x* ± SD	*x* ± SD
SAP (mmHg)	81 ± 20	111 ± 31*	95 ± 26	117 ± 33*	100 ± 28	120 ± 23
DAP (mmHg)	44 ± 10	44 ± 15	43 ± 12	35 ± 14*	35 ± 16	40 ± 11
MAP (mmHg)	57 ± 3	67 ± 6*	60 ± 2	64 ± 7*	57 ± 2	67 ± 5*
PPV (%)	15.68 ± 2.13	11.47 ± 1.24*	11.8 ± 1.01	10.85 ± 0.99*	11.13 ± 0.83	8.76 ± 3.89
CI (L min^−1^ M^2^)	2.09 ± 0.11	2.54 ± 0.18*	2.47 ± 0.17	2.49 ± 0.23	2.39 ± 0.26	2.8 ± 0.25
HR (min^−1^)	113 ± 8	96 ± 13*	109 ± 8	102 ± 13*	116 ± 7	99 ± 12*
SV (mL)	35 ± 5	51 ± 11*	43 ± 7	45 ± 10	38 ± 5	52 ± 12*
SVV (%)	18.45 ± 3.04	13.16 ± 3.58*	15.9 ± 4.32	11 ± 2.40*	11.13 ± 3.36	11.00 ± 1.78
Ea (mmHg mL^−1^)	2.11 ± 0.41	1.98 ± 0.35*	1.97 ± 0.37	2.33 ± 0.40*	2.38 ± 0.41	2.04 ± 0.33
Ees (mmHg mL^−1^)	1.42 ± 0.35	1.65 ± 0.33*	1.50 ± 0.3	1.54 ± 0.38	1.18 ± 0.20	1.63 ± 0.31*
Ea/Ees	1.56 ± 0.41	1.25 ± 0.36*	1.38 ± 0.44	1.63 ± 0.6*	2.10 ± 0.62	1.37 ± 0.71*
Pra (mmHg)	7.65 ± 1.39	8.53 ± 1.53*	8.55 ± 1.82	9.32 ± 1.76*	9.75 ± 1.16	6.6 ± 1.27*
Pmsa (mmHg)	12.98 ± 1.44	14.94 ± 1.50*	14.5 ± 1.79	15.24 ± 1.77*	15.41 ± 1.54	14.04 ± 2.53
Pvr (mmHg)	5.32 ± 0.49	6.41 ± 0.72*	5.95 ± 0.59	6.18 ± 0.78	5.66 ± 0.75	7.67 ± 1.65*
Eh (%)	0.41 ± 0.05	0.43 ± 0.06*	0.42 ± 0.06	0.41 ± 0.07	0.37 ± 0.04	0.54 ± 0.05*
LV SW (mmHg mL)	2581 ± 900	5299 ± 2349*	3580 ± 6120	5023 ± 2338*	6202 ± 2381	5845 ± 2356*
LV efficiency (%)	0.65 ± 0.12	0.74 ± 0.11*	0.71 ± 0.17	0.67 ± 0.12	0.65 ± 0.17	0.69 ± 0.06

Abbreviations as in Table 1, plus, *SAP* systolic arterial pressure, *DAP* diastolic arterial pressure, *PPV* pulse pressure variation, *SV* stroke volume, *SVV* stroke volume variation

*p < 0.05 with respect to the preceding resuscitation step

### Effect of VE

**Fig. 4:**
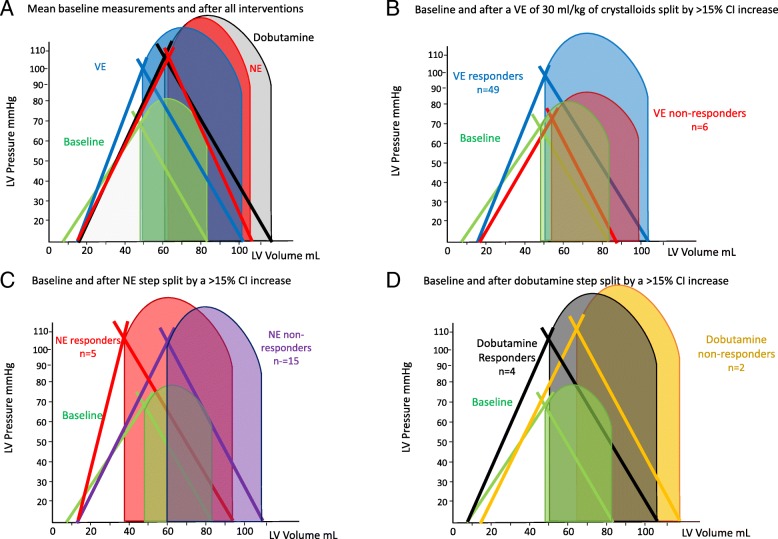
Group difference ventriculo-arterial coupling between **a** mean values for all patients at baseline, and following VE, under NE, if needed, and then, if needed, under dobutamine. **b** Group differences in relations between those patients who increased their CI > 15% to a VE of 30 mL/kg of crystalloids (responders) from non-responders. **c** Group differences in relations between those patients who increased their CI > 15% to NE (responders) from non-responders. **d** Group differences in relations between those patients who increased their CI > 15% to dobutamine (responders) from non-responders. The figure uses the same format, conventions, and abbreviations as Fig. 2 upper panel. LV left ventricle

**Fig. 5:**
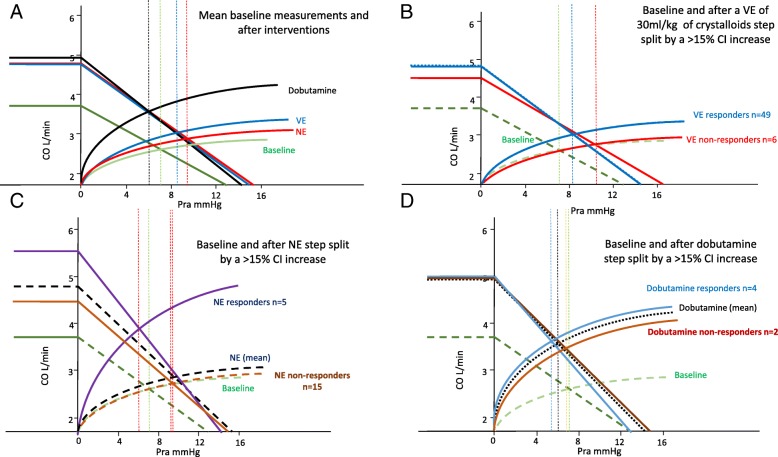
Group differences in venous return to cardiac output relations between **a** mean values for all patients at baseline, and following VE, under NE, if needed, and then, if needed under dobutamine. **b** Group differences in relations between those patients who increased their CI > 15% to a VE of 30 mL/kg of crystalloids (responders) from non-responders. **c** Group differences in relations between those patients who increased their CI > 15% to NE (responders) from non-responders. **d** Group differences in relations between those patients who increased their CI > 15% to dobutamine (responders) from non-responders. The figure uses the same format, conventions, and abbreviations as Fig. 2 bottom panel

**Fig. 6:**
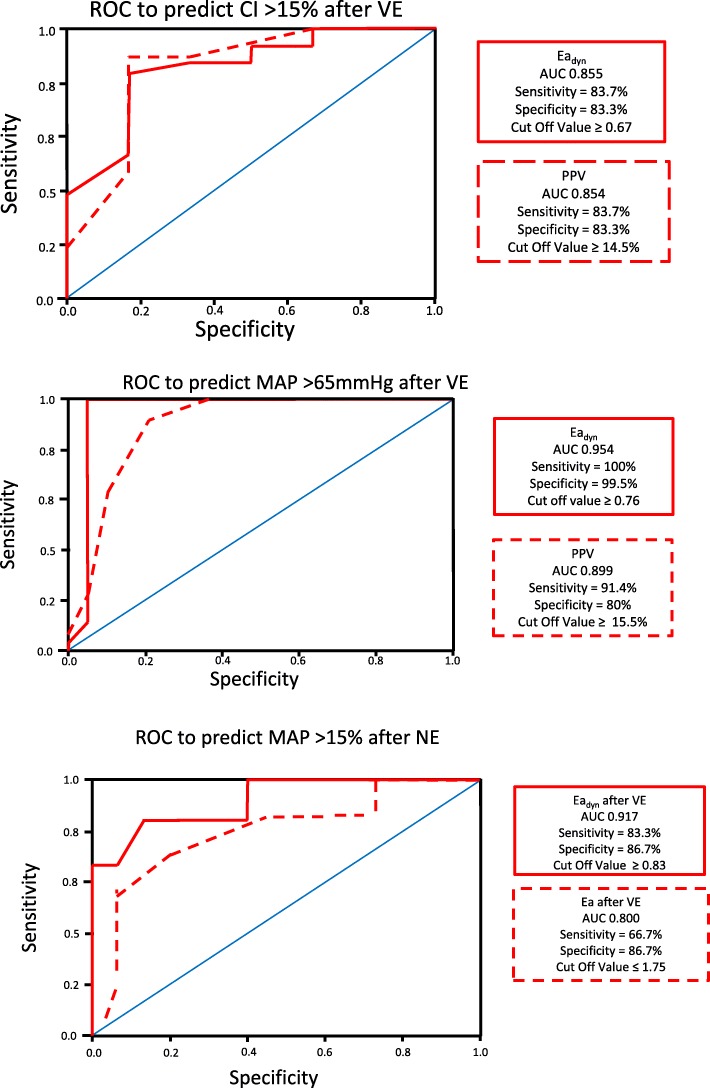
Receiver operator characteristic curse for each value for the most informative predictive variable and its threshold value to predict VE-induced changes in cardiac index (ΔCI) (upper panel) and MAP (ΔMAP) relative to baseline PPV and Ea_dyn_ (middle panel) for all patients and relation between ΔMAP in response to NE and PPV and Ea_dyn_ for all subjects given NE (lower panel)

The effects of VE on hemodynamic parameters are reported in Table 3. In all patients, both MAP and CI improved following VE, as well as cardiac contractility (Ees). Regarding Guytonian physiological parameters, fluid administration improved Pra, Pmsa, and Pvr in all patients. Moreover, both HR and Ea decreased, causing VAC to improve toward normal values, thus improving both LVeff and Eh. However, these hemodynamic changes were not uniformly observed across patients. Table 3 shows predefined subgroup analyses for CI and MAP responders and non-responders to each step of the protocol. Additional file 3: Figure S10 and S11 displays the CI responders (increase in CI > 15%) versus non-responders to a VE of 30 mL/kg of crystalloids, NE and dobutamine, respectively; Fig. 4 displays the Ees and Ea lines; and Fig. 5 displays the venous return differences between subgroups of responders and non-responders. CI increased by > 15% in 49 of 55 patients. The CI and MAP increases in response to VE and NE were proportional to the pre-treatment PPV and Ea_dyn_ values (Additional file 3: Figure S12, S13, S14). As expected, VE-induced CI increases were associated with decreases in both PPV and SVV. Similarly, CI responders had higher baseline Ea_dyn_, Ea, and Ees than CI non-responders. Importantly, a VE of 30 mL/kg of crystalloid also increased MAP > 65 mmHg in 35 patients (MAP responders). CI responders > 15% (Fig. 4) increased SV, arterial pressure, and Ees, whereas non-responders only increased LV end-diastolic volume and SV. MAP responders had higher PPV, SVV, and Ea_dyn_ than MAP non-responders. An Ea_dyn_ < 0.76 predicted MAP non-responders (Fig. 6), whereas baseline Ea > 1.9 mL mmHg^−1^ was a weak though also significant predictor of MAP responders. Finally, patients with more normal baseline Ea/Ees had greater increases in CI and MAP. Additional subgroup analyses are given in Additional file 1: Results.

**Table 3:** Responder vs. non-responder subgroup analysis: cardiovascular effects of resuscitation

MAP > 65 mmHg	Baseline		Volume expansion		Norepinephrine		Dobutamine	
Variable	Responders	Not responders	Responders	Not responders	Responders	Not responders	Responders	Not responders
Patients			35	20	12	11	6	2
SAP	85 ± 21	74 ± 18	121 ± 30*	95 ± 27*	138 ± 28*	100 ± 28*	124 ± 24	102 ± 25
DAP	43 ± 10	48 ± 7	44 ± 16	43 ± 12*	35 ± 13*	35 ± 16*	43 ± 11*	31 ± 15
MAP	57 ± 3	57 ± 3	70 ± 3*	60 ± 2*	70 ± 2*	57 ± 2*	70 ± 2*	60 ± 6
PPV	16.8 ± 1.1	13.7 ± 2.1	11.3 ± 1.3*	11.8 ± 1.1*	10.7 ± 1.1*	11.0 ± 0.9*	9.6 ± 3.5	6.3 ± 5.3
CI	2.09 ± 0.11	2.10 ± 0.11	2.59 ± 0.17*	2.47 ± 0.17*	2.60 ± 0.19*	2.39 ± 0.26	2.92 ± 0.04*	2.20 ± 0.31
HR	112 ± 8	115 ± 6	88 ± 8*	108 ± 8*	92 ± 5*	114 ± 8	93 ± 6*	115 ± 7
SV	34.8 ± 5.6	34.2 ± 3.8	54.9 ± 9.3*	42.9 ± 6.5*	54.1 ± 4.7*	37.5 ± 5.4	56.4 ± 8.0*	32.8 ± 6.2
SVV	17.2 ± 1.9	20.7 ± 3.4	11.6 ± 1.8*	15.9 ± 4.3*	11.1 ± 1.7*	11.0 ± 3.2*	11.0 ± 2.0	11.0 ± 1.4
Ea_dyn_	1.00 ± 0.13	0.67 ± 0.14	0.99 ± 0.15	0.80 ± 1.75*	0.98 ± 0.14	1.08 ± 0.38*	0.88 ± 0.44	0.60 ± 0.57
Ees	1.43 ± 0.39	1.40 ± 0.28	1.73 ± 0.31*	1.50 ± 0.30*	1.81 ± 0.25*	1.19 ± 0.19*	1.70 ± 0.09*	1.40 ± 0.71
Ea	2.20 ± 0.38	1.96 ± 0.42	1.98 ± 0.34*	1.97 ± 0.37	2.24 ± 0.37*	2.44 ± 0.44*	1.93 ± 0.12*	2.35 ± 0.63
Ea/Ees	1.62 ± 0.43	1.44 ± 0.36	1.74 ± 0.31*	1.38 ± 0.44	1.26 ± 0.28	2.12 ± 0.59*	1.14 ± 0.10*	2.05 ± 1.49
Pra	7.9 ± 1.2	7.3 ± 1.7	8.5 ± 1.4*	8.6 ± 1.8*	9.0 ± 2.1	9.9 ± 1.2*	6.8 ± 1.5*	6.5 ± 0.7
Pmsa	13.2 ± 1.2	12.6 ± 1.8	15.2 ± 1.3*	14.5 ± 1.8*	15.1 ± 2.1*	15.4 ± 1.5	15.4 ± 2.1	11.7 ± 1.7
Pvr	5.3 ± 0.5	5.3 ± 0.4	6.7 ± 0.5*	6.0 ± 0.6	6.7 ± 0.4*	5.7 ± 0.8	8.8 ± 0.8*	5.7 ± 0.8
Eh	0.41 ± 0.05	0.42 ± 0.57	0.44 ± 0.05*	0.42 ± 0.06	0.45 ± 0.06	0.37 ± 0.04*	0.57 ± 0.04*	0.49 ± 0.03
SW	2736 ± 963	2311 ± 723	6171 ± 2284*	3722 ± 1577*	6789 ± 1923*	3480 ± 1389	6411 ± 2131*	3019 ± 978
LV efficiency	0.67 ± 0.11	0.61 ± 0.12	0.74 ± 0.07*	0.73 ± 0.15*	0.68 ± 0.09	0.66 ± 0.15	0.70 ± 0.04	0.60 ± 0.11
CI > 15%	Baseline		Volume expansion		Norepinephrine		Dobutamine	
Variable	Responders	Not Responders	Responders	Not Responders	Responders	Not Responders	Responders	Not Responders
Patients			49	6	7	14	5	3
SAP	83 ± 20	66 ± 22	115 ± 29*	81 ± 34	103 ± 25	118 ± 34*	118 ± 32	122 ± 18
DAP	44 ± 10	51 ± 8	43 ± 15	51 ± 12	46 ± 1*	35 ± 14*	46 ± 14	35 ± 5
MAP	57 ± 3	56 ± 2	67 ± 6*	61 ± 4*	72 ± 2	62 ± 7	70 ± 2*	64 ± 8
PPV	16.0 ± 1.8	12.7 ± 2.3	11.4 ± 1.3*	11.8 ± 1.2	11.0 ± 1.4	11.1 ± 0.7*	11.3 ± 0.6	10.3 ± 0.6
CI	2.09 ± 0.12	2.07 ± 0.52	2.58 ± 0.16*	2.28 ± 0.12*	2.70 ± 0.17*	2.45 ± 0.23	2.86 ± 0.05*	2.70 ± 0.44*
HR	112 ± 7	119 ± 5	94 ± 12*	108 ± 11*	108 ± 13	105 ± 14	91 ± 8	102 ± 16
SV	34.5 ± 5.2	34.9 ± 2.9	51.4 ± 10.0*	43.2 ± 8.9*	47.4 ± 9.4	44.6 ± 9.9	54.2 ± 10.3*	50.7 ± 15.7
SVV	18.3 ± 3.1	19.7 ± 2.0	13.0 ± 3.5*	14.3 ± 4.2*	8.0 ± 2.8	11.3 ± 2.8*	11.7 ± 2.9	10.3 ± 0.6
Ea_dyn_	0.91 ± 0.20	0.65 ± 1.56	0.92 ± 0.20	0.89 ± 0.27	1.50 ± 0.70	1.04 ± 0.31*	1.01 ± 0.24	1.00 ± 0.10
Ees	1.44 ± 0.37	1.25 ± 0.15	1.67 ± 0.34*	1.48 ± 0.20*	1.70 ± 0.20	1.41 ± 0.35	1.70 ± 0.10*	1.43 ± 0.48
Ea	2.16 ± 0.37	1.70 ± 0.48	2.02 ± 0.33*	1.65 ± 0.34	1.83 ± 0.21	2.36 ± 0.36*	1.93 ± 0.15	2.27 ± 0.46
Ea/Ees	1.57 ± 0.39	1.44 ± 0.61	1.27 ± 0.36*	1.15 ± 0.38	1.09 ± 0.17	1.80 ± 0.62*	1.14 ± 0.12	1.82 ± 1.12
Pra	7.7 ± 1.4	7.2 ± 1.3	8.3 ± 1.4*	10.5 ± 1.2*	8.0 ± 1.7	9.3 ± 1.7*	5.8 ± 0.8*	7.3 ± 1.5 *
Pmsa	13.1 ± 1.4	12.4 ± 1.4	14.8 ± 1.5*	16.1 ± 1.3*	14.5 ± 1.4*	15.4 ± 1.8*	13.2 ± 1.9	15.4 ± 3.3
Pvr	5.3 ± 0.5	5.2 ± 0.3	6.5 ± 0.7*	5.6 ± 0.4*	6.5 ± 0.3*	6.1 ± 0.8	7.4 ± 1.6	8.1 ± 2.0
Eh	0.41 ± 1.47	0.42 ± 0.04	0.44 ± 0.50*	0.35 ± 0.03*	0.45 ± 0.07*	0.40 ± 0.06*	0.56 ± 0.06*	0.52 ± 0.04*
SW	2639 ± 902	2115 ± 808	5535 ± 2267	3373 ± 2294	4507 ± 1900	4965 ± 2415*	5932 ± 2804	5759 ± 2448
LV efficiency	0.65 ± 0.12	0.62 ± 0.08	0.73 ± 0.10*	0.80 ± 0.14*	0.77 ± 0.09	0.66 ± 0.12	0.71 ± 0.05	0.66 ± 0.06

Variable units and abbreviations as in Tables 1 and 2

*Statistically significant difference (*p* < 0.05) between mean variables values before and after each treatment split by positive response either in MAP > 65 mmHg (first part of the table) or in CI increase > 15% (second part of the table)

### Effect of NE

NE was given to the 20 of 55 patients who remained hypotensive post VE. NE increased MAP in 12 patients by 10 ± 2 mmHg and decreased MAP slightly in 8 by 2 ± 2 mmHg. NE was also associated with a decreased HR, PPV, and SVV, while Pra, Ea, Ea_dyn_, and Pmsa increased. VAC worsened to decoupled values like those seen at baseline, whereas both Eh and LVeff were unchanged. Those 12 patients in whom MAP increased > 65 mmHg (MAP responders) had higher pre-NE Ees and Ea values and lower HR than the MAP non-responders (Fig. 4c). An Ea_dyn_ > 0.83 and Ea > 1.75 predicted a MAP increase > 15% (Fig. 6). Not surprisingly, the CI response to NE was also variable, increasing in 5 and minimally decreasing in 4 patients. Interestingly, NE CO responders increased Ees improving VAC whereas non-responders did not (Fig. 4c).

### Effect of dobutamine

Dobutamine was given to 6 of the 8 patients whose MAP remained hypotensive following NE and VE. Dobutamine increased MAP, CI, and Ees causing VAC and Eh to improve. Both HR and Pra decreased, causing Pvr to increase, whereas neither PPV, SVV, Pmsa, nor LVeff changed. Pre-dobutamine Ea was higher in those whose MAP increased (Ea 2.73 ± 0.12 vs. 2.16 ± 0.37 mL mmHg^−1^, *p* = 0.02). Four of the 6 patients increased CO > 15%, and in those patients, Ees improved more than in the other 2 CO non-responders (Fig. 4d).

## Discussion

Our data demonstrate the heterogeneous cardiovascular profiles and responses to a SSG-defined resuscitation protocol in septic patients. Although our cohort received antibiotics within 2 h and VE within 3 h and completed the entire initial SSG protocol within 5 h, we still observed a 47% 30-day mortality underscoring the lethality of septic shock. Interestingly, survival was associated with admission lactate, BUN, and creatinine levels but not with initial hemodynamic measures including responsiveness to VE or NE. These data also illustrate four principal cardiovascular findings.

First, that in most patients, VE restores MAP to > 65 mmHg while simultaneously increasing CO, Ees, and Pmsa, resulting in an improved VAC, LVeff, and Eh. Presumably, the increasing MAP improved coronary perfusion increasing Ees. Although Ees is a load-independent parameter of LV contractility, LV contractility is also improved by restoring coronary perfusion pressure, which occurred in these patients (Figs. 2 and 4b). Our results are also comparable to a large database analysis of septic shock patients (*n* = 3686) that reported only two thirds of patients being volume responders [19]. We enrolled patients prior to any fluid resuscitation other than an initial 250–500 mL fluid used during initial catheter insertion, which may not be the case with patients referred from the emergency department or ward where prior boluses of crystalloid fluids may have already been given. Thus, the percent volume responder may be less in a previously resuscitated cohort. Our data are also consistent with prior findings that PPV predicts CO responses to VE in septic patients [14] and that Ea_dyn_ predicted the associated change in MAP in response to changing CO. [15] We found that individual patient MAP and CO responses to VE were variable, but accurately predicted by baseline PPV, SVV, and Ea_dyn_, suggesting that fluid resuscitation based on these dynamic measures may result in more efficient fluid resuscitation by giving less fluid to non-responders. Several patients did not increase the CO in response to VE. Non-responders may become volume overloaded, with its associated increase morbidity [20]. These data also support the clinical relevance of using dynamic measures to guide fluid resuscitation in septic patients as recommended by the SSG [3]. Similarly, as CO increased, both PPV and SVV decreased, showing that PPV and SVV trending can be used clinically to monitor dynamic changes in cardiac output in response resuscitation maneuvers in septic patients (Fig. 6, Additional file 3: Figure S7, S8), as previously suggested [14].

Second, NE increased Ea and MAP in most patients but did not achieve a MAP > 65 mmHg in a majority, consistent with prior studies of a variable response to norepinephrine in septic shock [21]. The pre-NE Ees, Ea, and VAC predicted which patients would increase most their CO, suggesting that baseline Ees and VAC define the LV response to NE. Importantly, NE infusion also induced ventriculo-arterial uncoupling to levels seen prior to resuscitation (Additional file 3: Figure S6) and also decreased LVeff, which, if sustained, might impair LV performance (Figs. 2 and 4c). This data support the recent finding that sustained vasopressors use of > 6 h to maintain a MAP > 75 mmHg in septic shock is associated with increased mortality [22]. However, it is unclear what level of VAC, its duration, or level of exogenous catecholamines are necessary to create this detrimental effect. Recently, animal studies demonstrate that NE can impair LV ejection by increasing the magnitude of arterial pressure reflected waves during ejection, which also becomes manifest as VAC uncoupling without increasing coronary perfusion pressure [23]. As was also seen in patients with postoperative vasoplegia [11], we observed that only patients with higher Ees and normalized VAC increased CO during NE (Fig. 4), presumably because they can tolerate the increased afterload.

Third, when dobutamine was added, VAC normalized while CO increased in all subjects. MAP simultaneously increased by a small but clinically significant amount (Fig. 4a, d). These data underscore the impact that exogenous inotropic support may improve contractility in septic patients who may be affected by septic cardiomyopathy [13]. These data also demonstrate the combined effect of dobutamine on both the arterial (Ea) and venous (Pmsa) circulations. Our data demonstrate that combined LV contractility and arterial tone processes, linked to venous return and LV performance [18], interact to define the arterial pressure and CO response to VE, NE, and dobutamine. Similar pooled cardiovascular parameter analyses to ours in future studies may increase our understanding of how other vasoactive and inotropic therapies, like vasopressin, angiotensin II or methylene blue infusions, and the use of low-dose corticosteroids, impact cardiovascular performance [21].

Fourth, at all times (VE, NE, dobutamine), changes in CO were proportional to changes in Pvr, independent of Pmsa changes (Fig. 3). These data are consistent with previous studies in postoperative surgical patients [24], stable septic patients being weaned from NE [25], and animals given dobutamine [7] and also validate that the Guytonian determinants of cardiovascular homeostasis [26] remain operative in septic patients during initial resuscitation. Specifically, VE and NE increased Pmsa and the subsequent change in CO was proportional to the change in Pvr. If NE decreased CO, it was because Pra increased more than Pmsa, as quantified by a decreasing Eh, whereas dobutamine, though decreasing both Pmsa and Pra, decreased Pra more, such that Pvr increased. Thus, modulating effective circulating blood volume with fluids and vasopressors is only one way to increase CO. Dobutamine increased CO by decreasing Pra. Importantly, septic patients still hypotensive during NE increased their MAP to dobutamine while restoring normal VAC, underscoring VAC as a fundamental determinant of MAP and CO responses in septic shock, as shown by others [11, 12, 18].

### Limitations

The study has several limitations. First, we did not personalize resuscitation therapy but used a defined SSG resuscitation protocol. Still, our findings agree with previous studies on VE responders [27] and support the use of dynamic parameters to predict CO and MAP responsiveness to VE [14, 15, 25]. Second, we only measured these cardiovascular variables during the initial 5-h resuscitation interval. Although we hypothesize that these parameters would continue to be predictive of cardiovascular response, other processes might decrease the predictive value of these measures. Still, during the initial rescue phase of septic shock [28], these parameters appear robust. Third, Ea, Ees, and Pmsa were calculated using formulae rather than measuring them directly using more invasive means. Thus, to the extent that the assumptions made to create these measures are inaccurate in severe sepsis, our measurement accuracy will degrade. For example, Ea is highly dependent on heart rate. Still, in our subjects, though tachycardic were unchanged during resuscitation. We doubt that other calculation inaccuracies exist because all these measures have been validated against more definitive measures in animal models of septic shock [29], and in humans during both septic shock [25] and post-cardiopulmonary bypass vasoplegia [30]. End-systolic pressure was estimated as 0.9∙systolic arterial pressure measured at the radial artery. Systolic arterial pressure may be altered in sepsis as the measuring cite moves distal from the aorta. Still, in animal models, the subsequent change in systolic arterial pressure with resuscitation was similar across all measuring sites, meaning that the directional changes in calculated Ees and Ea would remain accurate independent of monitoring loci. Although we calculated Pmsa using formulae based on both CO and Pra [8], the computational components for Pra were an order of magnitude less than the observed changes in Pvr. Furthermore, we validated our Pmsa measures on endotoxic dogs using direct measures of Pms by transient stop flow during step-wise volume infusion and withdrawal, demonstrating an excellent correlation [29]. Furthermore, our Ea and Ees values compare to those of other studies [11, 12, 18]. Finally, we estimated CO and SV using an uncalibrated arterial pressure waveform analysis algorithm (Mostcare®). To the extent that its assumptions become inaccurate during NE and dobutamine infusion, then the accuracy of these data will degrade. However, others have shown that the Mostcare® device follows CO in septic shock patients given NE [31, 32]. Furthermore, as listed in the methods, we checked the accuracy of the Mostcare® CO estimates with echocardiographic estimates at each step and found the two methods consistent.

### Clinical perspective

From the clinical perspective, several points become clear. First, it is impossible to know a priori if a septic patient who presents with hypotension fits the Sepsis-3 criteria for septic shock [33] without first performing VE, as fully two thirds of our patients reversed their hypotension with the initial fluid resuscitation alone. Second, it is not clear if separating patients into either sepsis or septic shock is clinically relevant because the mortality rate in our cohort and their levels of end-organ injury was unaffected by this separation. Third, individualized fluid resuscitation based on the use of dynamic markers of fluid responsiveness allow for both accurate prediction of subsequent absolute change in cardiac output and, if followed continually, the actual changes in cardiac output over time. Fourth, although norepinephrine restores or sustained arterial pressure in septic shock patients, it does so at the expense of the heart, by selectively increasing Ea and decoupling LV ejection power from afterload. Thus, these data argue for limiting use of NE to initially restoring MAP and organ perfusion and then tapering it off once stable.

## Conclusion

Initial fluid resuscitation based on SSG restores MAP and CI in most patients, while simultaneously restoring VAC and LVeff. However, individual patient responses vary widely. Similarly, the responses to NE in those persistently hypotensive or to subsequent dobutamine were also variable, but predictable based on their pre-treatment physiologic state. Finally, NE induced ventriculo-arterial decoupling which potentially may cause myocardial damage, if persistent. The bedside determination of Ea, Ees, and VAC plus dynamic parameters of volume responsiveness (e.g., PPV, SVV) and arterial tone (Ea_dyn_) is useful to both predict the responses to therapy and understand the differences in hemodynamic response among septic shock patients during resuscitation.

## Additional files

Additional file 1: Supplemental Methods and Results (DOCX 25 kb)

Additional file 2: **Table S1.** Individual patient characteristics. (DOCX 13 kb)

Additional file 3: **Figure S1.** Relation between individual values of cardiac output (CO) over steps in the protocol: baseline, volume expansion (VE), plus norepinephrine (+NE), and plus dobutamine. **Figure S2.** Relation between individual values of mean arterial pressure (MAP) over steps in the protocol: baseline, volume expansion (VE), plus norepinephrine (+NE), and plus dobutamine. **Figure S3.** Relation between individual values of left ventricular end-systolic elastance (Ees) over steps in the protocol: baseline, volume expansion (VE), plus norepinephrine (+NE), and plus dobutamine. **Figure S4.** Relation between individual values of arterial elastance (Ea) over steps in the protocol: baseline, volume expansion (VE), plus norepinephrine (+NE), and plus dobutamine. **Figure S5.** Relation between individual values of mean systemic pressure analogue (Pmsa) over steps in the protocol: baseline, volume expansion (VE), plus norepinephrine (+NE), and plus dobutamine. **Figure S6.** Relation between individual values of ventriculo-arterial coupling (VAC) from baseline to volume expansion (VE) to plus norepinephrine (+NE) with mean ± SD for each step shown in blue. Values above 1.35 reflect uncoupling and values below 1.35 reflect normal VAC. **Figure S7.** Baseline to Volume Expansion relation between change in CO (DCO) and either pre-volume expansion pulse pressure variation (PPV) or dynamic arterial elastance (Ea_dyn_). These data relate to the receiver operating characteristic results in Fig. 4. **Figure S8.** Baseline to volume expansion relation between change in mean arterial pressure (DMAP) and either pre-volume expansion pulse pressure variation (PPV) or dynamic arterial elastance (Ea_dyn_). These data relate to the receiver operating characteristic results in Fig. 4. **Figure S9.** Volume Expansion to Norepinephrine relation between change in mean arterial pressure (DMAP) and either pre-norepinephrine arterial elastance (Ea) or dynamic arterial elastance (Ea_dyn_). These data relate to the receiver operating characteristic results in Fig. 4. (PPTX 395 kb)
