# Supplementary material for: Cardiovascular determinants of resuscitation from sepsis and septic shock
Source: Crit Care. 2019 Apr 15;23:118. doi: 10.1186/s13054-019-2414-9 (PMC6466803; doi:10.1186/s13054-019-2414-9)
Supplement: Supplementary file 1 — Supplemental Methods and Results (DOCX 25 kb) [file 13054_2019_2414_MOESM1_ESM.docx]

**Supplemental Content**

***Expanded Methods*:**

Left ventricular (LV) end-systolic elastance (Ees) was calculated by using the single-beat method (E_es(sb_)) as reported by Chen et al. (5). Normalized Ees at arterial end-diastole (E_Nd(est)_) was measured according to the following formula:

E_es(sb)_ = [P_d_ – (E_Nd(est)_ x P_s_ x 0.9)]/[SV x E_Nd(est)_]

E_Nd(avg)_ = Σa_i_ x t_Nd_

i = 0

E_Nd(est)_ = 0.0275 - 0.165 x EF + 0.3656 x (P_d_/P_es_) + 0.515 x E_Nd(avg)_

where ai values are 0.35695, −7.2266, 74.249, −307.39, 684.54. −856.92, 571.95 and −159.1 for i = 1 to i = 7, respectively. The t_Nd_ value was determined by the ratio of pre-ejection period (R-wave to flow onset) to total systolic period (R-wave to end-flow), with the time of onset and termination of flow-defined Doppler. Systolic blood pressure (Ps) and diastolic blood pressure (Pd) were measured invasively. EF is ejection fraction.

Vo is also estimated by the single beat method as:

Vo = [ESP – (E_es_ x ESV)]/E_es_.

where ESP is end-systolic pressure and ESV is end-systolic volume. This is derived by rearranging the E_es_ to:

ESP= (E_es_ x ESV) + b

where b = ESP - (E_es_ x ESV)

Mean systemic pressure analogue (Pmsa) as an estimate of mean systemic pressure was calculated by the method of Parkin and Leaning (21), as recently automated by Vos et al. (Vos JJ et al J Clin Monit Comput. 2018 Feb;32(1):73-80. doi: 10.1007/s10877-017-9990-5. Epub 2017 Feb 16.) according to the following formula:

$$Pmsa=a\cdot Pra+b\cdot MAP+c\cdot CO$$

Where a and b are dimensionless constants (a+b = 1, typically a = 0.96 and b = 0.04) and c has dimension of resistance and is calculated as:

$$c=0.038\cdot(94.17+0.193\cdot age)/(4.5\cdot[{0.9}^{\left( age-15 \right)}]\cdot0.007184\cdot[\mathrm{height}^{0.725}]\cdot[\mathrm{weight}^{0.425}]$$

Where mean arterial pressure (MAP) and right atrial pressure (Pra) are in mmHg, cardiac output (CO) in L·min^-1^, age in years, height in cm and weight in Kg.

Derived parameters include the pressure gradient for venous return (Pvr) as Pmsa-Pra, and global cardiac performance as Pvr·Pmsa^-1^.

***Expanded Results*:**

The effects of VE, NE and dobutamine on individual patient CO, MAP, Ees, Ea, Pmsa and VAC are shown in figures E1-6, respectively.

Additional statistical analyses performed on these data include Receiver-Operator Characteristic curve analysis of specific threshold value predictors of >10%, >15% and >20% CI increase or >15% MAP increase are reported below.

**Predicting MAP and CO increases in response to VE.**

CI increase by >10% had a higher Ea_dyn_ (diff 34.7% mean 0.59±0.78 in non-responders v. 0.90±0.20 in responders, p=0.01), Ea (diff 36.6% mean 1.37±0.12 in non-responders v. 2.16±0.42 in responders, p<0.0005), VAC (diff 37.4% mean 1.0±0.8 in non-responders v. 1.6±0.4 in responders, p=0.01), and PPV (diff 26.6% mean 11.7±1.2 in non-responders v. 15.9±1.9% in responders, p=0.002). No other measured separated responders from non-responders at a >10% CI increase.

CI increase by >15% again had higher Ea_dyn_ (diff 28.5% mean 0.65±0.16 in non-responders v. 0.91±0.20 in responders, p=0.003), Ea (diff 21.4% mean 1.70±0.48 in non-responders v. 2.16±0.37 in responders, p=0.003), Ees (diff 13.2% mean 1.25±0.15 in non-responders v. 1.44±0.37 in responders, p=0.03), HR (diff 6.3% mean 119.3±5.1 in non-responders v. 112.3±7.5 in responders, p=0.03), and PPV (diff 21.0% mean 12.7±2.3 in non-responders v. 16.0±1.8 in responders, p=0.003). No other measured separated responders from non-responders at a >15% CI increase.

CI increased by >20% had higher Ea_dyn_ (diff 14.0% mean 0.80±0.24 in non-responders v. 0.93±0.18 in responders, p=0.03), Ea (diff 16.0% mean 1.89±0.37 in non-responders v. 2.24±0.37 in responders, p=0.03), CI (diff 3.1% mean 2.13±0.08 in non-responders v. 2.07±0.12 in responders, p=0.04), and PPV (diff 11.7% mean 14.5±2.6% in non-responders v. 16.4±1.5 in responders, p=0.03). No other measured separated responders from non-responders at a >20% CI increase.

Patients whose MAP increased to >65 mmHg in response to VE displayed higher baseline PPV (diff 18.9% mean 13.7±2.1 in non-responders v. 16.8±1.0 in responders p<0.0005), SVV (diff 20.1%, mean 20.7±3.4 in non-responders v. 17.2±1.9 in responders, p<0.0005) and Ea_dyn_ (diff 33.3%, mean 0.67±0.14 in non-responders v. 1.03±0.13 in responders, p<0.0005). These baseline predictors of an increase in MAP persisted when only directional increases in MAP of >10%, >15% or >20% were made. Data for >10% were Baseline PPV (diff 13.9% mean 14.1±2.1 in non-responders v. 16.331.8 in responders, p<0.0005), SVV (diff 21.2%, mean 21.1±3.6 in non-responders v. 17.4±2.0 in responders, p=0.001) and Ea_dyn_ (diff 29.6% 0.68±0.15 in non-responders vs 0.96±0.17 in responders, p<0.0005).

**Predicting MAP responses to NE**

Patients whose MAP >65 mmHg in response to NE displayed higher post-VE Ees (diff 20.0% mean 1.33±0.21 in non-responders v. 1.67±0.32 in responders, p=0.014), HR (diff 8.1% mean 112.6±6.7 in non-responders v. 104.2±8.2 in responders, p=0.021). No other measures separated responders from non-responders for an increase in MAP to >65 mmHg.

Patients whose MAP increased by >10% had higher values for VAC (diff 32.2% mean 1.54±0.39 in non-responders vs 1.17±0.40 in responders, p=0.044), SVV (diff 28.9% mean 17.33±3.63 in not responders v. 13.44±4.39 in responders, p=0.039) and Ea_dyn_ (diff 25.8% mean 0.69±0.11 in not responders vs 0.93±0.28 in responders, p=0.033). No other measures separated hypotensive patients responders from non-responders for an increase in MAP > 10%.

Patients whose MAP increased by >15% had higher values for Ea_dyn_ (diff 35.0% mean 0.69±0.13 in non-responders v. 1.06±0.26 in responders, p<0.0005), Ea (diff 23.9% mean 2.13±0.37 in non-responders v. 1.71±0.33 in responders, p=0.030), and SVV (diff 50.8% mean 17.3±3.8 in non-responders v. 11.5±2.3 in responders, p=0.003). No other measures separated hypotensive patients responders from non-responders for an increase in MAP > 15%.

Patients whose MAP increased by >20% had higher values for SVV (diff 48.9% mean 16.2±4.3 in non-responders vs 11.0±0 in responders, p<0.0005) and Pvr (diff 20.7% mean 6.0±0.5 in not responders v. 5.0±0.40 in responders, p=0.012). No other measures separated hypotensive patients responders from non-responders for an increase in MAP > 20%.

**Predicting CI responses to NE**

## Patients whose CI increased by 15% a higher MAP (diff 4.5% mean 60.1±2.1 in non-responders v. 63.0±0 in responders, p=0.011), but that was the only factor that differentiate this subgroup.

**Electronic supplement figures**

Figure E1. Relation between individual values of cardiac output (CO) over steps in the protocol: baseline, volume expansion (VE), plus norepinephrine (+NE), and plus dobutamine.

Figure E2. Relation between individual values of mean arterial pressure (MAP) over steps in the protocol: baseline, volume expansion (VE), plus norepinephrine (+NE), and plus dobutamine.

Figure E3. Relation between individual values of left ventricular end-systolic elastance (Ees) over steps in the protocol: baseline, volume expansion (VE), plus norepinephrine (+NE), and plus dobutamine.

Figure E4. Relation between individual values of arterial elastance (Ea) over steps in the protocol: baseline, volume expansion (VE), plus norepinephrine (+NE), and plus dobutamine.

Figure E5. Relation between individual values of mean systemic pressure analogue (Pmsa) over steps in the protocol: baseline, volume expansion (VE), plus norepinephrine (+NE), and plus dobutamine.

Figure E6. Relation between individual values of ventriculo-arterial coupling (VAC) from baseline to volume expansion (VE) to plus norepinephrine (+NE) with mean±SD for each step shown in blue. Values above 1.35 reflect uncoupling and values below 1.35 reflect normal VAC.

Figure E7. Baseline to Volume Expansion relation between change in CO (ΔCO) and either pre-volume expansion pulse pressure variation (PPV) or dynamic arterial elastance (Ea_dyn_). These data relate to the receiver operating characteristic results in figure 6.

Figure E8. Baseline to Volume Expansion relation between change in mean arterial pressure (ΔMAP) and either pre-volume expansion pulse pressure variation (PPV) or dynamic arterial elastance (Ea_dyn_). These data relate to the receiver operating characteristic results in figure 6.

Figure E9. Volume Expansion to Norepinephrine relation between change in mean arterial pressure (ΔMAP) and either pre-norepinephrine arterial elastance (Ea) or dynamic arterial elastance (Ea_dyn_). These data relate to the receiver operating characteristic results in figure 6.
