# Supplementary material for: Cardiovascular determinants of resuscitation from sepsis and septic shock
Source: Crit Care. 2019 Apr 15;23:118. doi: 10.1186/s13054-019-2414-9 (PMC6466803; doi:10.1186/s13054-019-2414-9)
Supplement: Supplementary file 2 — Table S1. Individual patient characteristics. (DOCX 13 kb) [file 13054_2019_2414_MOESM2_ESM.docx]

**Table S1. Individual patient characteristics**

| **Patient** | **Outcome** | **Sepsis etiology** | **SOFA** | **SAPS II** | **NE max dose mcg/kg/min** | **Dobu max dose mcg/kg/min** |
| --- | --- | --- | --- | --- | --- | --- |
| 1 | 0 | Pneumonia | 22 | 70 | 0 | 0 |
| 2 | 1 | Pneumonia | 12 | 51 | 0 | 0 |
| 3 | 0 | Mediastinitis | 15 | 66 | 0 | 0 |
| 4 | 0 | Bacteremia | 13 | 82 | 1.43 | 0 |
| 5 | 1 | Pneumonia | 11 | 47 | 1.00 | 0 |
| 6 | 0 | Pneumonia | 21 | 92 | 1.46 | 0 |
| 7 | 0 | Bacteremia | 16 | 76 | 0.90 | 0 |
| 8 | 1 | Pneumonia | 12 | 55 | 0.80 | 0 |
| 9 | 1 | Pneumonia | 13 | 56 | 1.00 | 12 |
| 10 | 0 | Bacteremia | 18 | 77 | 1.00 | 15 |
| 11 | 0 | Urosepsis | 22 | 84 | 1.00 | 15 |
| 12 | 0 | MV Endocarditis | 24 | 78 | 0.97 | 11 |
| 13 | 0 | MV Endocarditis | 15 | 65 | 1.00 | 15 |
| 14 | 0 | Peritonitis | 23 | 87 | 0 | 0 |
| 15 | 1 | Pneumonia | 12 | 55 | 0 | 0 |
| 16 | 1 | Bacteremia | 11 | 46 | 0 | 0 |
| 17 | 1 | Pneumonia | 16 | 65 | 0 | 0 |
| 18 | 1 | Urosepsis | 11 | 45 | 0 | 0 |
| 19 | 0 | Pneumonia | 26 | 76 | 0 | 0 |
| 20 | 1 | TV Endocarditis | 5 | 45 | 0 | 0 |
| 21 | 1 | Urosepsis | 12 | 44 | 0 | 0 |
| 22 | 0 | Endoplastitis | 19 | 76 | 0 | 0 |
| 23 | 1 | Peritonitis | 5 | 42 | 0 | 0 |
| 24 | 0 | Bacteremia | 12 | 76 | 0 | 0 |
| 25 | 1 | Bacteremia | 6 | 55 | 0 | 0 |
| 26 | 1 | Empyema | 22 | 65 | 0.40 | 0 |
| 27 | 0 | Erysipelas | 25 | 78 | 0 | 0 |
| 28 | 0 | Pneumonia | 29 | 67 | 0.50 | 0 |
| 29 | 0 | Mediastinitis | 37 | 80 | 0 | 0 |
| 30 | 0 | Peritonitis | 31 | 77 | 0 | 0 |
| 31 | 0 | Pneumonia | 17 | 67 | 0 | 0 |
| 32 | 1 | Pneumonia | 6 | 32 | 0 | 0 |
| 33 | 0 | Pneumonia | 24 | 67 | 0 | 0 |
| 34 | 0 | Pneumonia | 23 | 68 | 0.80 | 0 |
| 35 | 1 | Pneumonia | 5 | 36 | 0.50 | 0 |
| 36 | 1 | Pneumonia | 12 | 54 | 0.45 | 0 |
| 37 | 0 | Pneumonia | 32 | 76 | 0 | 0 |
| 38 | 1 | Pneumonia | 7 | 45 | 0.35 | 0 |
| 39 | 0 | LRTI | 12 | 67 | 1.00 | 10 |
| 40 | 0 | Pneumonia | 15 | 73 | 1.00 | 8 |
| 41 | 1 | Bacteremia | 23 | 77 | 1.00 | 13 |
| 42 | 1 | Bacteremia | 7 | 53 | 0.40 | 0 |
| 43 | 1 | Bacteremia | 7 | 66 | 0 | 0 |
| 44 | 0 | Pneumonia | 14 | 78 | 0 | 0 |
| 45 | 1 | Pneumonia | 12 | 45 | 0 | 0 |
| 46 | 1 | Pneumonia | 11 | 65 | 0 | 0 |
| 47 | 1 | Pneumonia | 15 | 43 | 0 | 0 |
| 48 | 1 | Peritonitis | 22 | 76 | 0 | 0 |
| 49 | 1 | Encephalitis | 12 | 77 | 0 | 0 |
| 50 | 1 | MV Endocarditis | 9 | 38 | 0 | 0 |
| 51 | 0 | Pneumonia | 23 | 68 | 0 | 0 |
| 52 | 0 | Pneumonia | 21 | 59 | 0 | 0 |
| 53 | 1 | Bacteremia | 14 | 48 | 0 | 0 |
| 54 | 1 | Pneumonia | 18 | 56 | 0 | 0 |
| 55 | 1 | Bacteremia | 11 | 44 | 0 | 0 |
|  |  |  |  |  |  |  |
| Outcome: alive=1, dead=0; MV= mitral valve; TV= tricuspid valve; LRTI= low respiratory tract infection | | | | | | |
