# Supplementary material for: Cardiovascular determinants of resuscitation from sepsis and septic shock
Source: Crit Care. 2019 Apr 15;23:118. doi: 10.1186/s13054-019-2414-9 (PMC6466803; doi:10.1186/s13054-019-2414-9)
Supplement: Supplementary file 3 — Figure S1. Relation between individual values of cardiac output (CO) over steps in the protocol: baseline, volume expansion (VE), plus norepinephrine (+NE), and plus dobutamine. Figure S2. Relation between individual values of mean arterial pressure (MAP) over steps in the protocol: baseline, volume expansion (VE), plus norepinephrine (+NE), and plus dobutamine. Figure S3. Relation between individual values of left ventricular end-systolic elastance (Ees) over steps in the protocol: baseline, volume expansion (VE), plus norepinephrine (+NE), and plus dobutamine. Figure S4. Relation between individual values of arterial elastance (Ea) over steps in the protocol: baseline, volume expansion (VE), plus norepinephrine (+NE), and plus dobutamine. Figure S5. Relation between individual values of mean systemic pressure analogue (Pmsa) over steps in the protocol: baseline, volume expansion (VE), plus norepinephrine (+NE), and plus dobutamine. Figure S6. Relation between individual values of ventriculo-arterial coupling (VAC) from baseline to volume expansion (VE) to plus norepinephrine (+NE) with mean ± SD for each step shown in blue. Values above 1.35 reflect uncoupling and values below 1.35 reflect normal VAC. Figure S7. Baseline to Volume Expansion relation between change in CO (DCO) and either pre-volume expansion pulse pressure variation (PPV) or dynamic arterial elastance (Eadyn). These data relate to the receiver operating characteristic results in Fig. 4. Figure S8. Baseline to volume expansion relation between change in mean arterial pressure (DMAP) and either pre-volume expansion pulse pressure variation (PPV) or dynamic arterial elastance (Eadyn). These data relate to the receiver operating characteristic results in Fig. 4. Figure S9. Volume Expansion to Norepinephrine relation between change in mean arterial pressure (DMAP) and either pre-norepinephrine arterial elastance (Ea) or dynamic arterial elastance (Eadyn). These data relate to the receiver operating char [file 13054_2019_2414_MOESM3_ESM.pptx]

## Slide 1
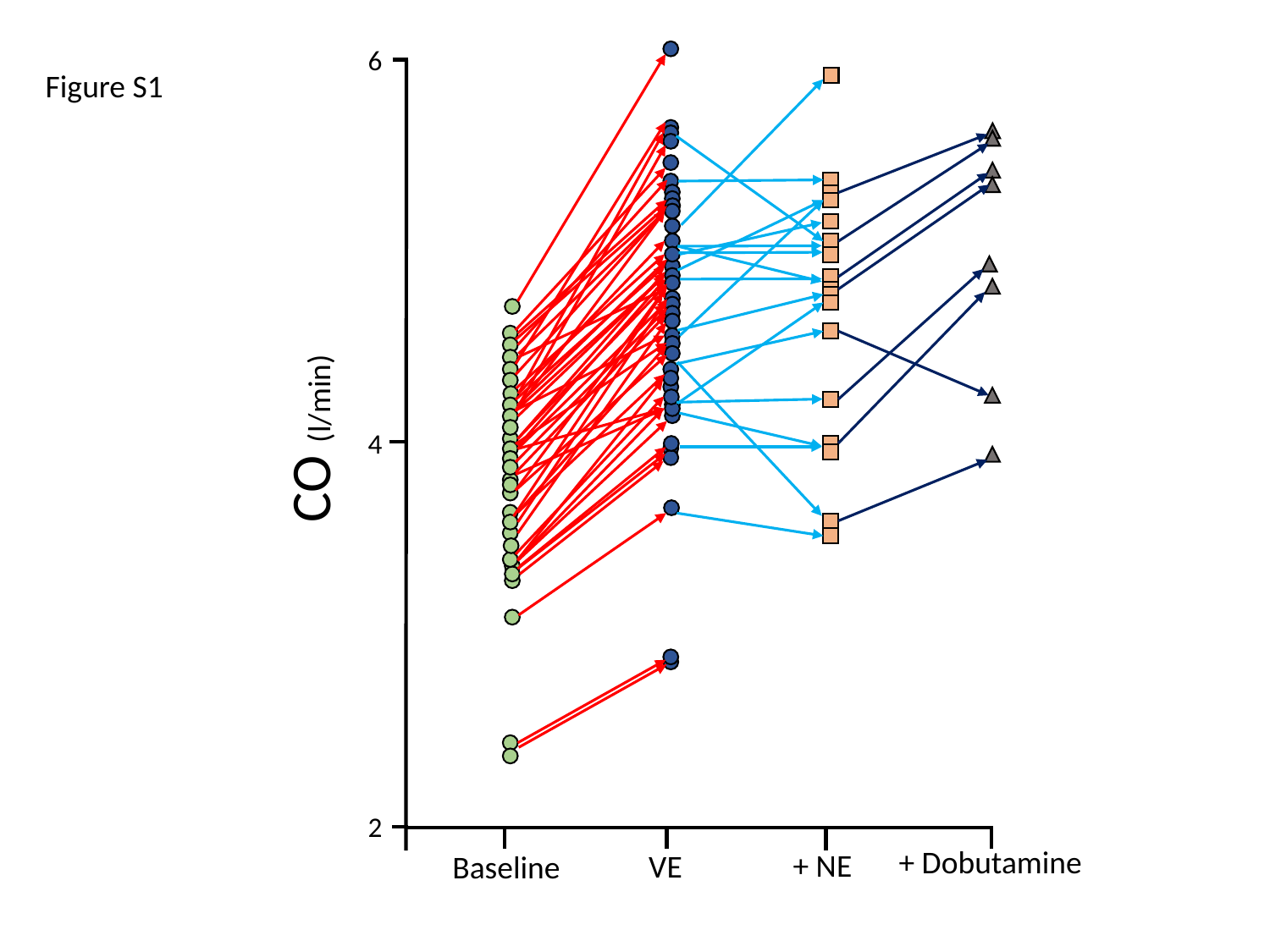

6
Figure S1
CO (l/min)
4
2
+ Dobutamine
+ NE
VE
Baseline

## Slide 2
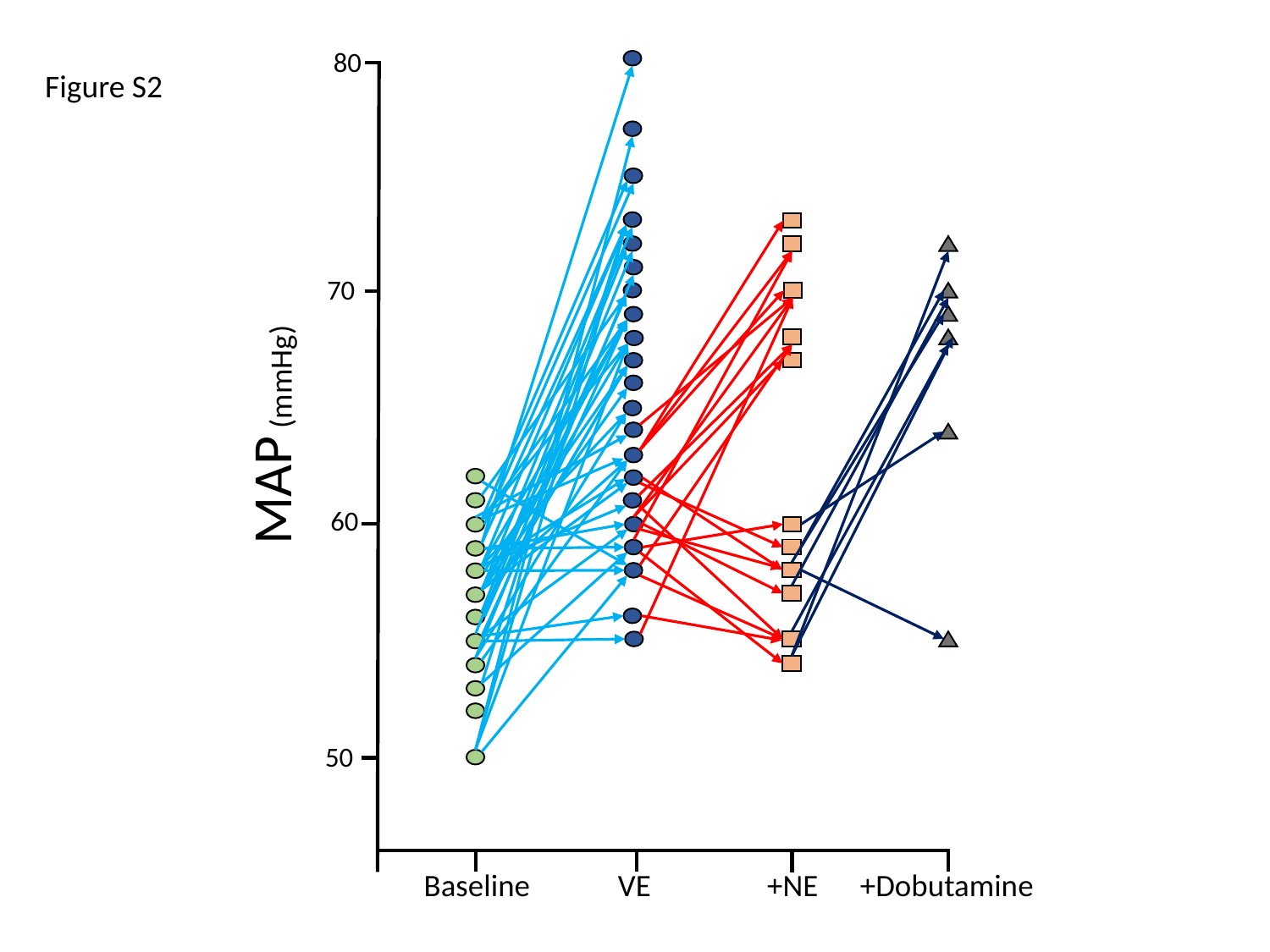

Figure S2

## Slide 3
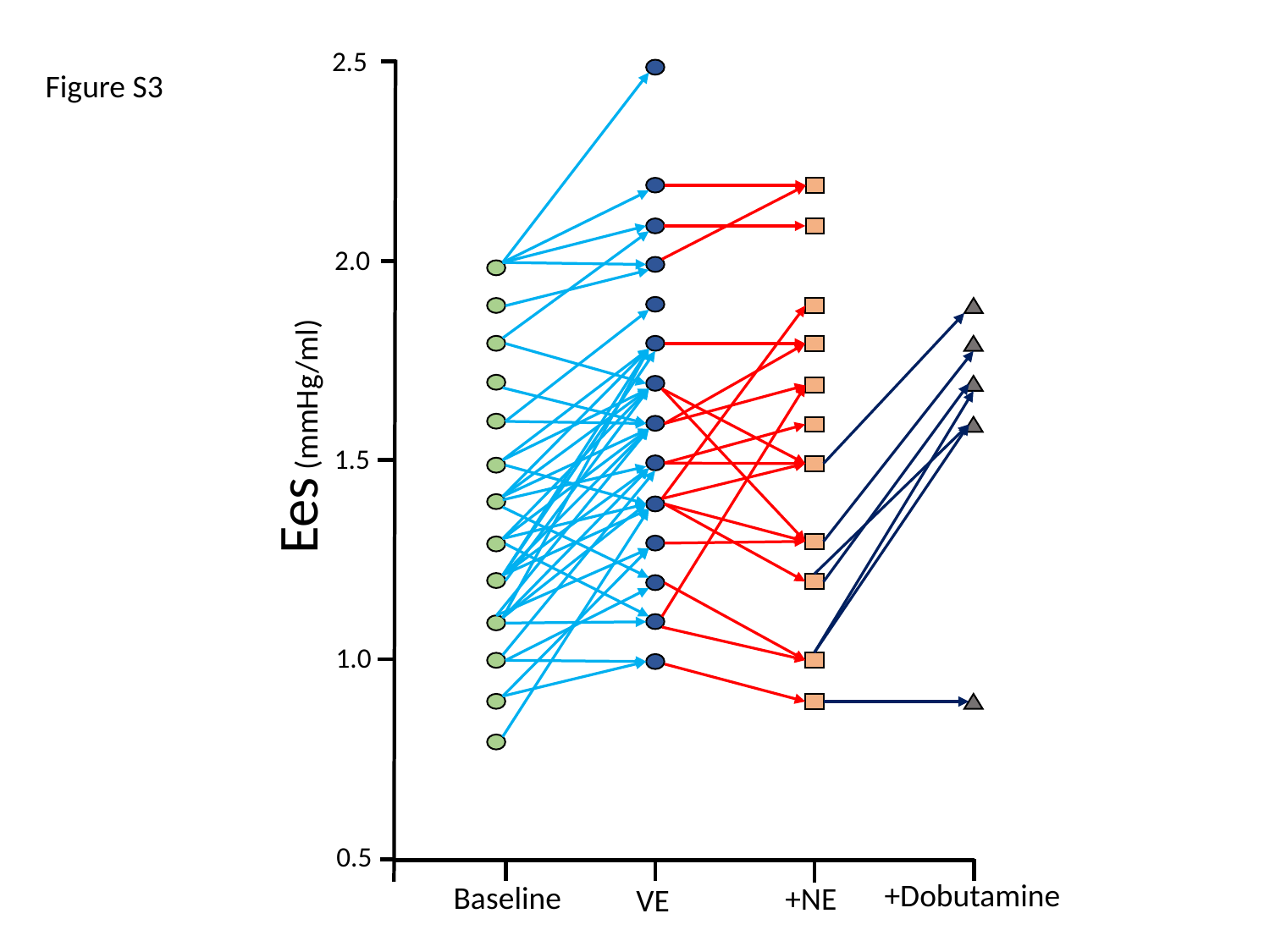

Figure S3

## Slide 4
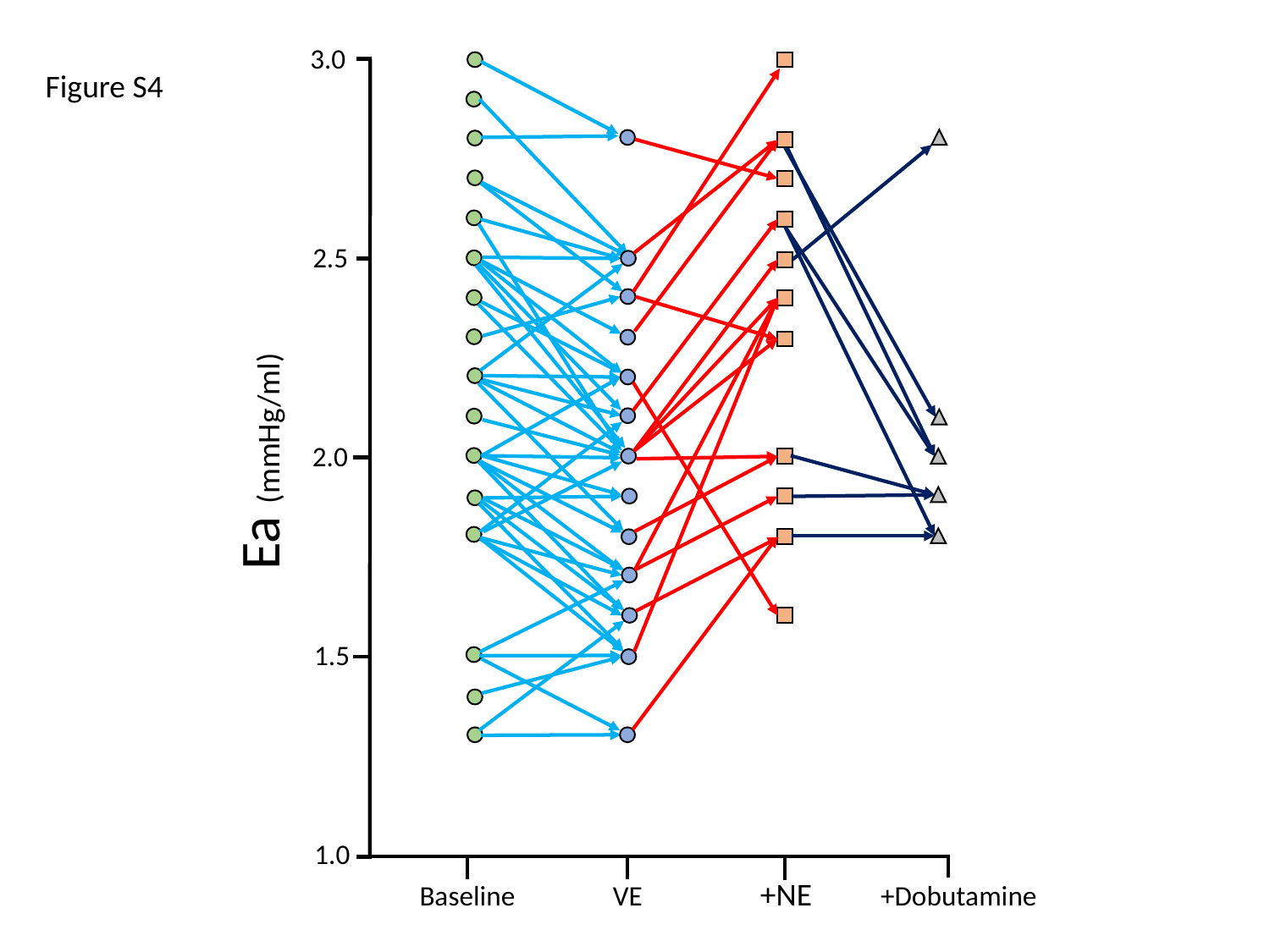

Figure S4

## Slide 5
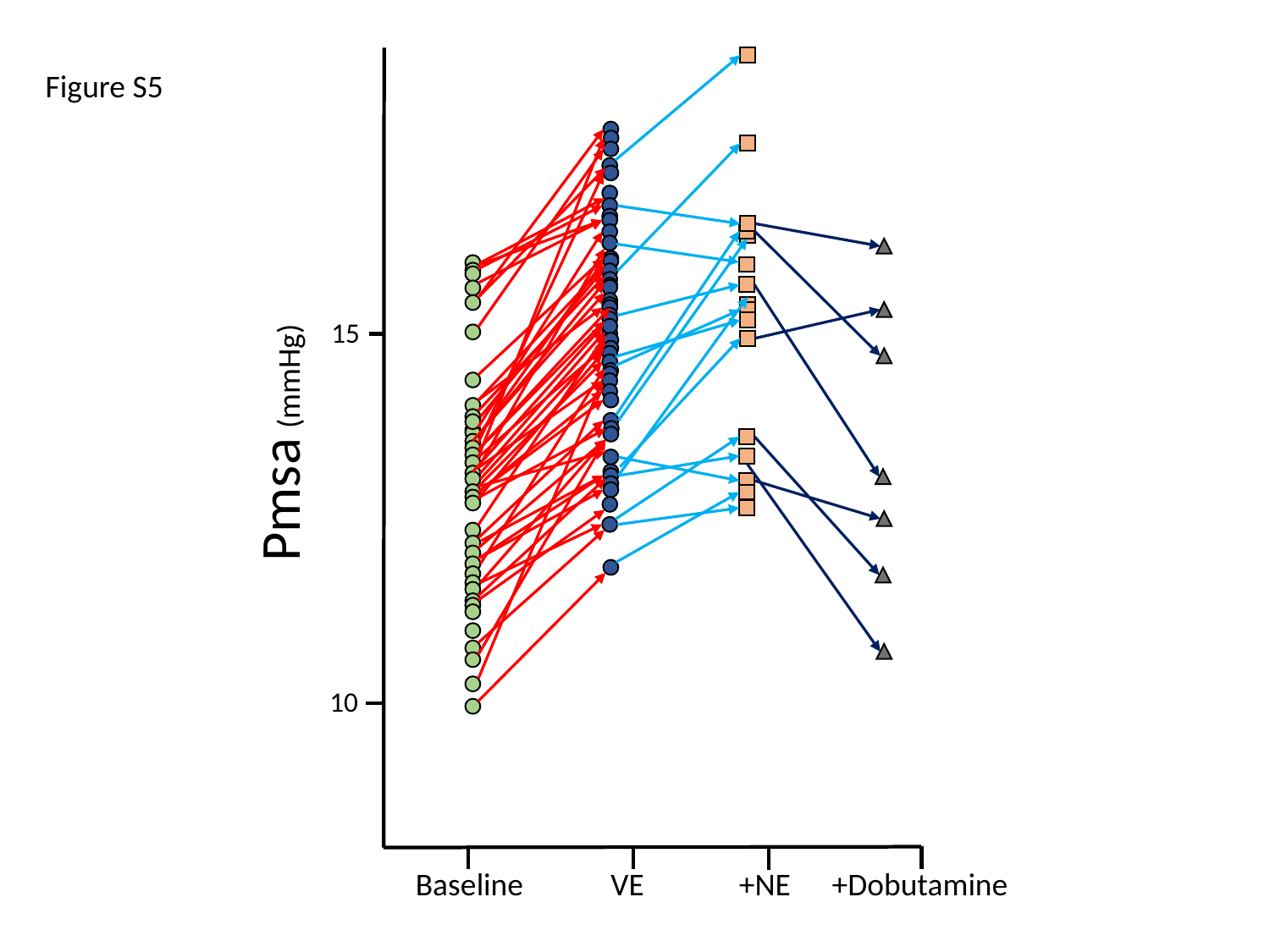

Figure S5

## Slide 6
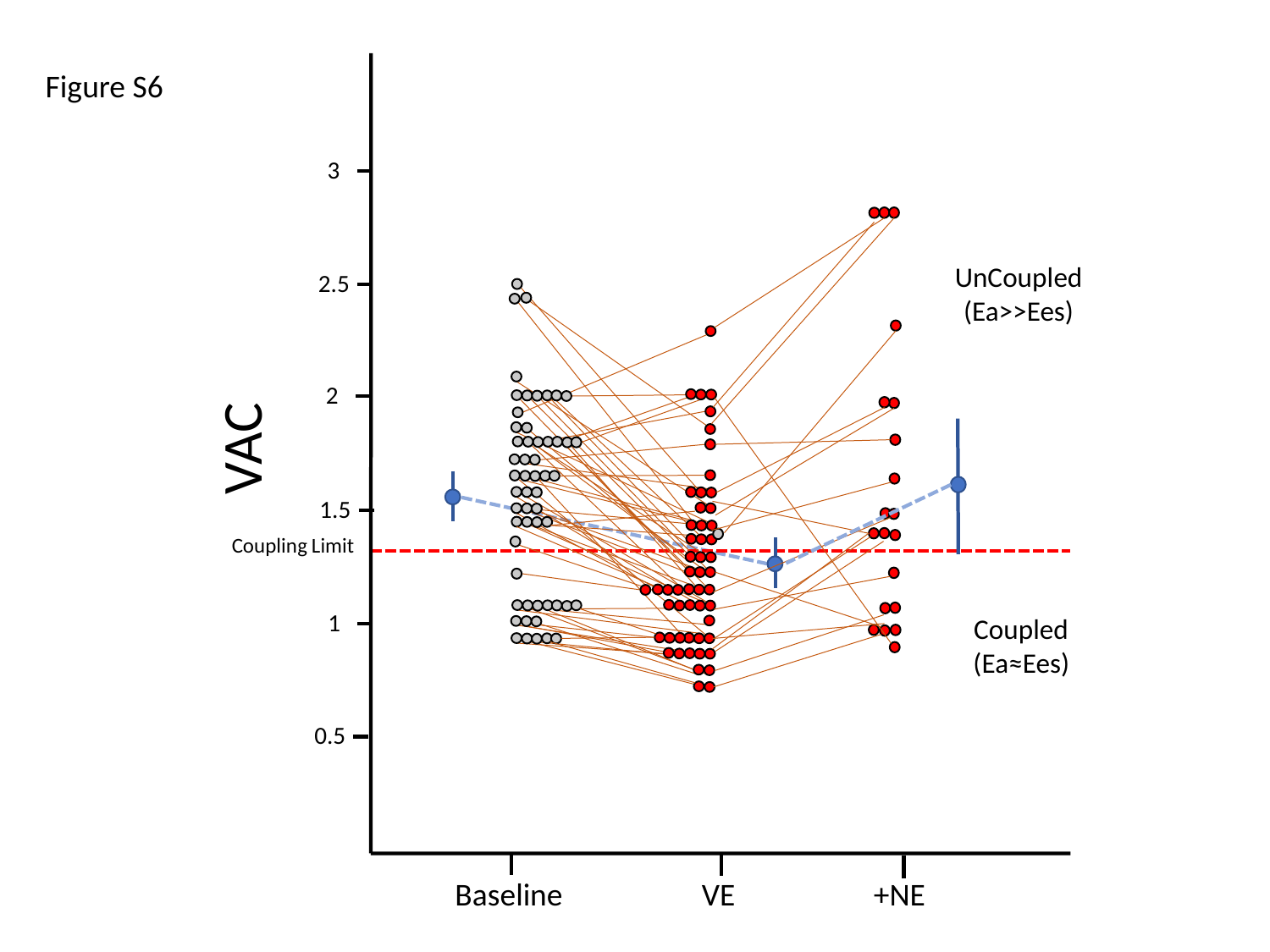

Figure S6

## Slide 7
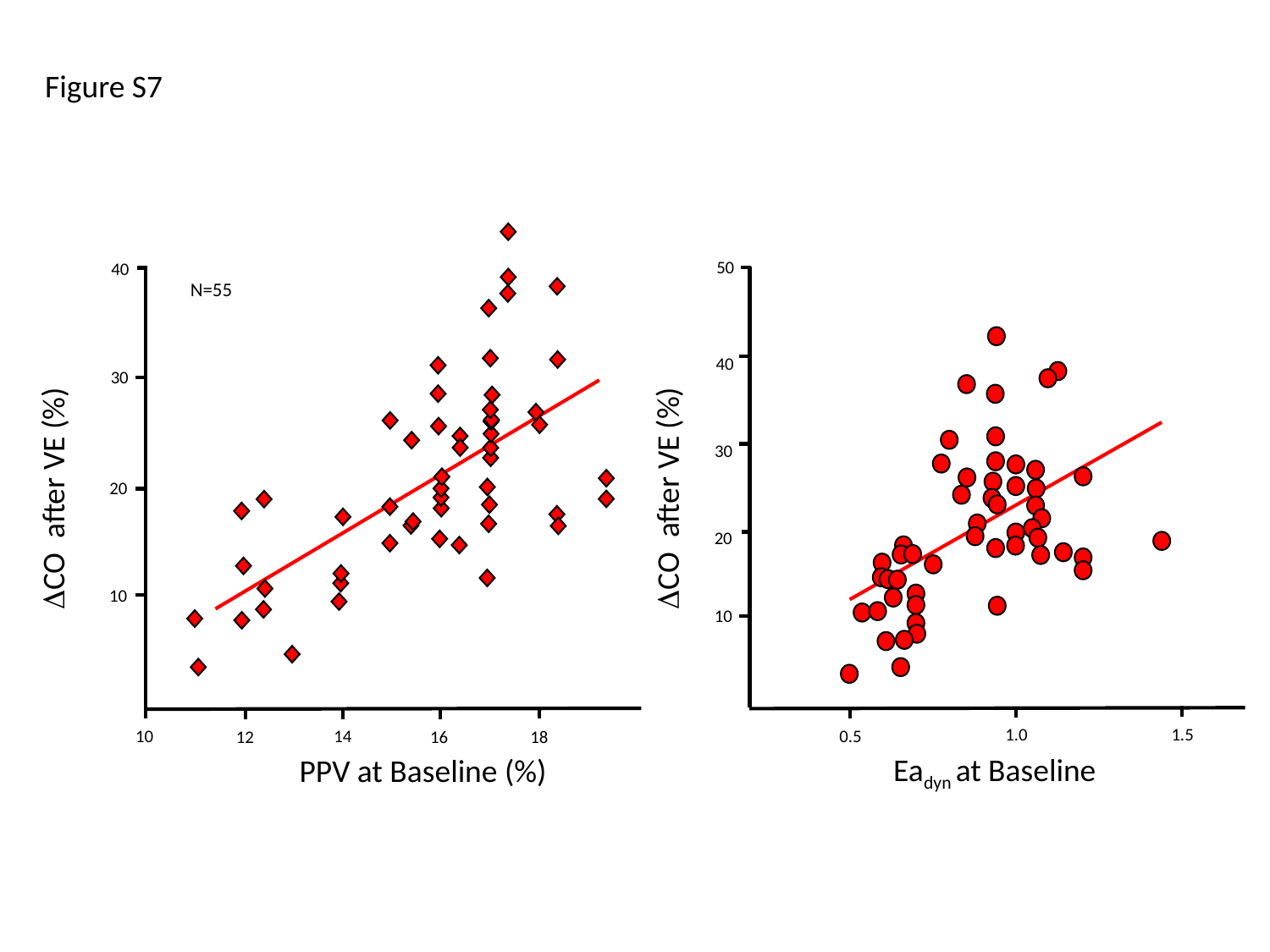

Figure S7
50
40
N=55
40
30
30
DCO after VE (%)
DCO after VE (%)
20
20
10
10
1.0
1.5
0.5
14
10
16
12
18
Eadyn at Baseline
PPV at Baseline (%)

## Slide 8
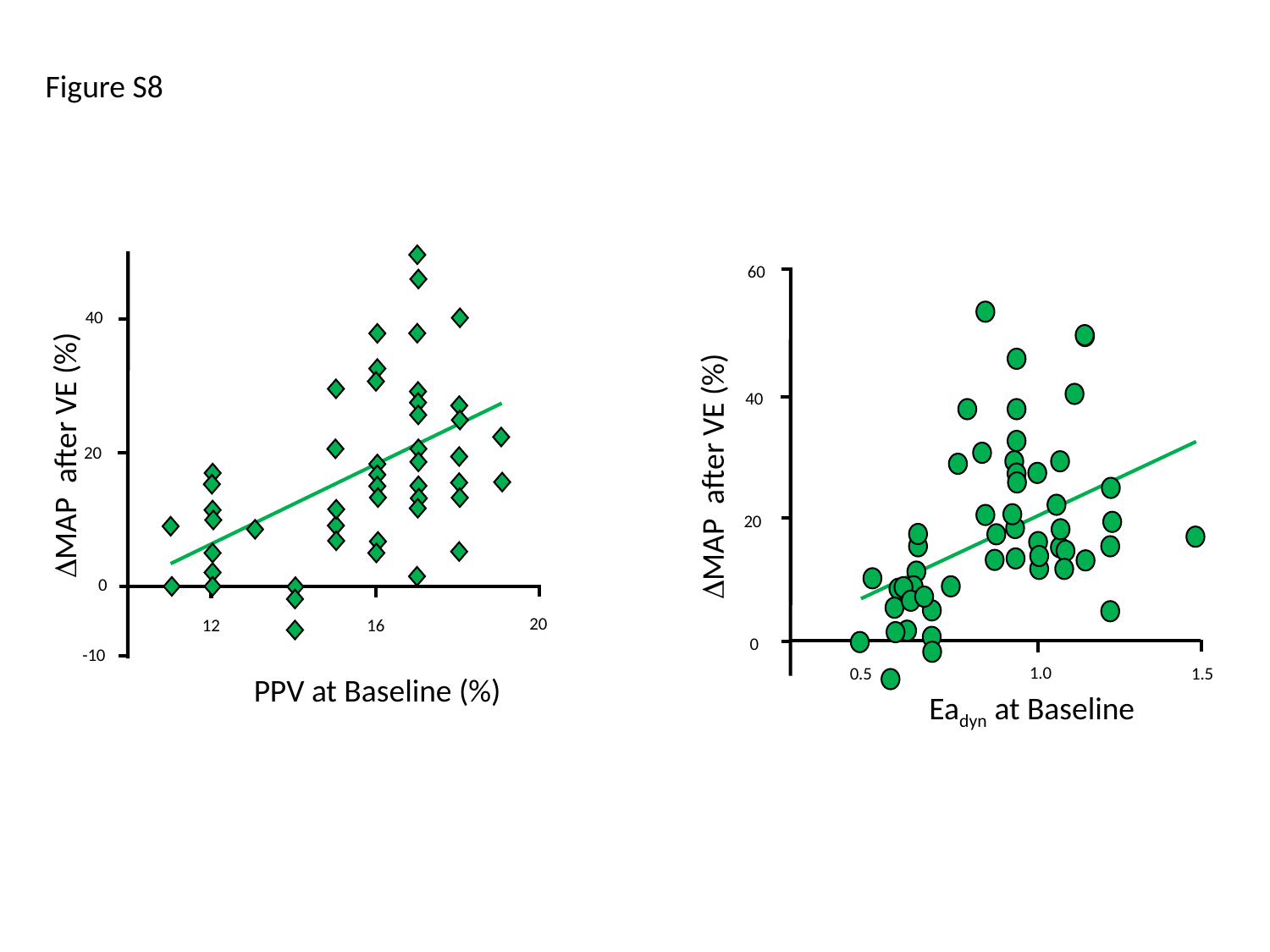

Figure S8
60
40
40
DMAP after VE (%)
DMAP after VE (%)
20
20
0
20
12
16
0
-10
1.0
0.5
1.5
PPV at Baseline (%)
Eadyn at Baseline

## Slide 9
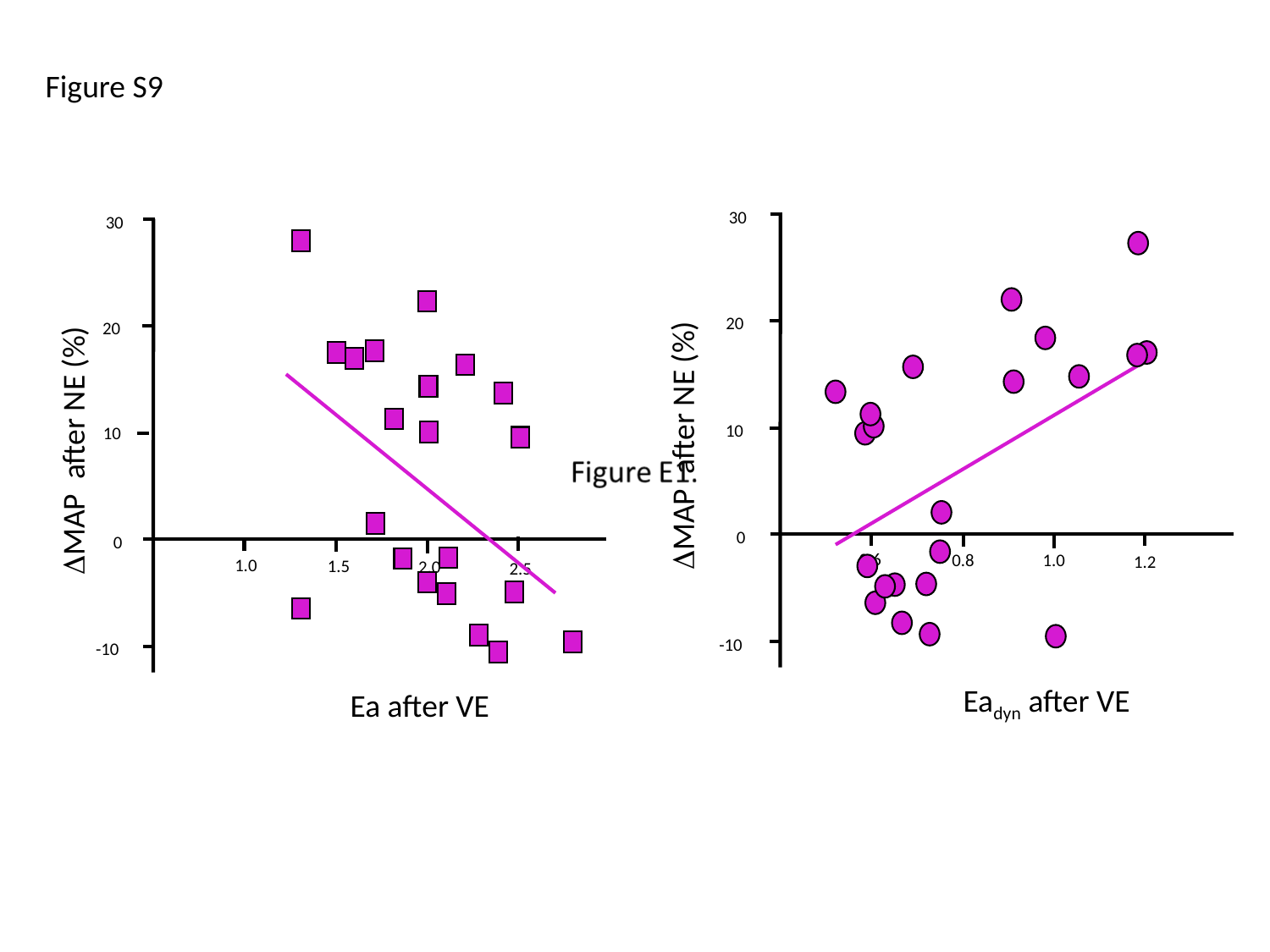

Figure S9
30
30
20
20
DMAP after NE (%)
DMAP after NE (%)
10
10
0
0
0.6
0.8
1.0
1.2
1.0
1.5
2.0
2.5
-10
-10
Eadyn after VE
Ea after VE
